# Editorial: Modern Endovascular Therapy for Peripheral Arterial Disease

**DOI:** 10.3389/fcvm.2021.651489

**Published:** 2021-03-19

**Authors:** Romaric Loffroy, Salah D. Qanadli

**Affiliations:** ^1^Centre Hospitalier Regional Universitaire De Dijon, Dijon, France; ^2^Centre Hospitalier Universitaire Vaudois (CHUV), Lausanne, Switzerland

**Keywords:** research, peripheral arterial disease, endovascular therapies, intravascular ultrasound, percutaneous transluminal angioplasty, atherectomy

Atherosclerotic disease is nowadays one of the most important concerns of modern medicine because it is a major cause of morbidity and mortality worldwide. Atheromatous lesions of the lower limbs, known as peripheral arterial disease (PAD), can significantly affect the quality of life and lead to disability in a high proportion of patients. The number of peripheral catheter-based procedures for endovascular treatment of PAD, which represent a promising alternative therapeutic option, has increased over the past years. Indeed, several techniques have been improved over time and now contribute to modern concepts of endovascular repair. This special issue aimed to provide a comprehensive overview of this lively research field by gathering contributions covering imaging and technical endovascular features, clinical studies and practical applications.

The study of Rotzinger et al. reported the results of a randomized controlled study assessing different image acquisition techniques using computed tomography angiography (CTA) in lower limbs. The authors prospectively compared in 60 patients three image acquisition techniques: the “traditional” anterograde method (SA), the adaptive anterograde method (AA), and the retrograde acquisition method (RA). Quantitative and qualitative analysis were performed. In terms of qualitative and quantitative analysis, AA gave better results than SA and RA, reporting the best vascular enhancement in a high percentage of patients. The presence of stenotic or occlusive lesions did not significantly impact the attenuation values among all levels and acquisition methods. The authors showed that AA should be recommended as the method of choice, especially in the presence of aneurysms which had a significant impact on the vessel attenuation in AA (high attenuation) and RA (low attenuation) at the iliac artery (*P* = 0.03 and 0.04) and femoral artery levels (*P* = 0.02 and <0.01). Alternatively, the authors concluded that SA may be useful in patients with renal failure, since the test bolus can be avoided. The spreading availability of rapid computed tomography scans could likely overcome the limits of RA.

In this issue,  Pescatori et al. conducted a systematic review of the different re-entry devices currently available for aortoiliac occlusive disease. Indeed, standard endoluminal revascularization fails in treating occlusions at the aortoiliac level in almost 20% of patients. In this setting, subintimal recanalization may be an option, but failure is reported in about 25% of patients as well. Several devices have been developed over the last decade, in order to facilitate the cross back within the true lumen, when conventional subintimal recanalization does not work or is at risk of occlusion of important collaterals. Pioneer, outback, offroad, and enteer catheters were described in this overview and the applications for those devices were fully revised.

In the same way,  Saucy et al. emphasized the importance of vessel preparation for optimizing endovascular therapy of infrainguinal lesions and provided a review of the different techniques allowing to gain lumen before vascular stenting or angioplasty using drug-coated technology. Over the last 5 years, the use of self-expandable nitinol stents has permitted endovascular repair of long atherosclerotic lesions, allowing significant improvement of clinical outcomes as compared to plain balloon angioplasty. Drug-eluting balloons have also emphasized the ability of this technology to decrease the incidence of restenosis and to drive target repair. However, calcified lesions and elastic recoil of the arterial vessel wall are still considered as risk factors for early restenosis and failed procedure. Therefore, vessel preparation using focused atherectomy catheters has shown promising results in modifying vessel compliance and debulking obstructive calcifications in order to improve long-term patency, as nicely described in this review.

More in detail,  Loffroy et al. conducted a review of safety, efficacy and outcomes of one of those debulking devices in patients with acute and subacute ischemia of the lower limbs. The authors described the use of Rotarex®S catheter as a percutaneous pure rotational mechanical atherectomy plus thrombectomy (MATH) device in such a setting as compared to rheolytic thrombectomy devices. MATH is a minimally invasive technique for rapidly recanalizing thrombotic lesions whatever the type and age of thrombus is. Indeed, many patients considered as chronic patients may present with critical limb ischemia, with occlusive lesions containing thrombus triggered by underlying atheromatous disease. The authors showed that MATH provides different advantages over surgery and catheter-directed thrombolysis, with faster revascularization, lower invasiveness, and the capacity to treat the underlying atherosclerotic lesions promptly. It is also associated with a lower rate of complications such as bleedings and avoids the need for intensive care unit hospitalization. Cost-effectiveness of the routine use of this device was also discussed here, with the existing evidence being favorable.

Last, Loffroy et al. discussed the place of IVUS in PAD through a systematic overview, notably the main applications of IVUS technique during interventions including angioplasty, stenting, recanalization of complete occlusion, evaluation of residual stenosis and stent delivery and expansion, and atherectomy procedure. Over recent years, IVUS has gained acceptance as a useful adjunctive tool to conventional angiography in a high proportion of catheter-based interventions for PAD. IVUS allows evaluation of the plaque features, artery diameter, and the presence of dissections. In addition, the authors showed that IVUS has the capability to accurately guide the best choice of appropriate percutaneous angioplasty method, guide the deployment of several catheters and devices, and evaluate the immediate outcomes of any vascular procedure. Again, despite the cost of this catheter, most of studies evaluated in this review showed that the use of IVUS during endovascular interventions can be cost-effectiveness.

Several factors have driven the evolution of percutaneous interventions for PAD over the last decade (Qanadli). First, the development of disruptive innovations such as drug delivery technology. Second, the improvement of evidence-based techniques to guide treatments. Many modalities for image-guided therapies have become available and useful as adjunctive tools such as IVUS, image fusion, three-dimensional imaging, robotics, and virtual or enhanced reality. Last, the development of specific catheters for vessel preparation such as atherectomy and thrombectomy devices allowed to get back to an old concept called “leave nothing behind” that has never been so relevant to optimize outcomes. Without any doubt, the next decade should allow active research in percutaneous interventions with promising perspectives and progress in all these aspects, especially with the use of artificial intelligence. In this setting, it is of the utmost importance to remember that being innovative should in no way prevent vascular specialists and interventional radiologists from remaining dedicated clinicians.

## Author Contributions

Each author has participated sufficiently in this manuscript to take public responsibility for its content. RL and SQ: conception and design of the study, or acquisition of data, or analysis, interpretation of data, drafting the article or revising it critically for important intellectual content, and final approval of the version to be submitted. Both authors contributed to the article and approved the submitted version.

## Conflict of Interest

The authors declare that the research was conducted in the absence of any commercial or financial relationships that could be construed as a potential conflict of interest.

